# Medical Chemistry

**Published:** 1879-12

**Authors:** 


MEDICAL CHEMISTRY, Including the outlines of organic and Physiological Chemistry, Based, in part, upon Biche's Manual de Chimie. By C. Gilbert Wheeler, Professor of Chemistry in the University of Chicago, etc. Second and Revised Edition. Wm. Wood & Co., N. Y. 1880.

The medical student requires varied modes of instruction to insure the essential comprehension of chemistry in the space of time allotted to the regular collegiate course in this country. To the medical beginner there is an intuitive and undefined vagueness about this branch of the medical science that renders a satisfactory understanding of its principles difficult to gain. Elementary works like the one before us are calculated to overcome this difficulty in a manner, and we take pleasure in recommending them to that class who need them most.

